# Chronic dysglycemia and risk of SARS‐CoV‐2 associated respiratory failure in hospitalized patients

**DOI:** 10.1111/aas.13982

**Published:** 2021-10-11

**Authors:** Susanne Rysz, Malin Jonsson Fagerlund, Claire Rimes‐Stigare, Emma Larsson, Francesca Campoccia Jalde, Johan Mårtensson

**Affiliations:** ^1^ Department of Perioperative Medicine and Intensive Care Karolinska University Hospital Stockholm Sweden; ^2^ Department of Medicine Solna Karolinska Institutet Stockholm Sweden; ^3^ Department of Physiology and Pharmacology Karolinska Institutet Stockholm Sweden; ^4^ Department of Molecular Medicine and Surgery Karolinska Institutet Stockholm Sweden

**Keywords:** dysglycemia, respiratory failure, severe Covid‐19

## Abstract

**Background:**

Diabetes is common among patients with severe acute respiratory syndrome coronavirus 2 (SARS‐CoV‐2)‐induced respiratory failure. We aimed to investigate the relationship between different stages of chronic dysglycemia and development of respiratory failure in hospitalized SARS‐CoV‐2 positive patients.

**Methods:**

In this retrospective observational study, we included 385 hospitalized SARS‐CoV‐2 positive patients at Karolinska University Hospital, Sweden with an HbA1c test obtained within 3 months before admission. Based on HbA1c level and previous diabetes history, we classified patients into the following dysglycemia categories: prediabetes, unknown diabetes, controlled diabetes, or uncontrolled diabetes. We used multivariable logistic regression analysis adjusted for age, sex, and body mass index, to assess the association between dysglycemia categories and development of SARS‐CoV‐2‐induced respiratory failure.

**Results:**

Of the 385 study patients, 88 (22.9%) had prediabetes, 68 (17.7%) had unknown diabetes, 36 (9.4%) had controlled diabetes, and 83 (21.6%) had uncontrolled diabetes. Overall, 299 (77.7%) patients were admitted with or developed SARS‐CoV‐2‐induced respiratory failure during hospitalization. In multivariable logistic regression analysis compared with no chronic dysglycemia, prediabetes (OR 14.41, 95% CI 5.27–39.43), unknown diabetes (OR 15.86, 95% CI 4.55–55.36), and uncontrolled diabetes (OR 17.61, 95% CI 5.77–53.74) was independently associated with increased risk of SARS‐CoV‐2‐induced respiratory failure.

**Conclusion:**

In our cohort of hospitalized SARS‐CoV‐2 positive patients with available HbA1c data, prediabetes, undiagnosed diabetes, and poorly controlled diabetes were associated with a markedly increased risk of SARS‐CoV‐2‐associated respiratory failure.

**Editorial Comment**

The study findings show that identification of patients with chronic dysglycemia and quantification of their premorbid control is clinically important since patients with undiagnosed dysglycemia or poor diabetes control are at particular risk of developing respiratory complications when infected with SARS‐CoV‐ 2. It was also found that that HbA1c measurement identifies a significant proportion of SARS‐CoV‐2 positive patients with prediabetes or previously unknown diabetes.

## 1 INTRODUCTION

Severe acute respiratory syndrome coronavirus 2 (SARS‐CoV‐2) has challenged healthcare systems around the world over the past year. Large case numbers and homogeneity in characteristics and presentation has allowed us to rapidly acquire some understanding of the disease. There remain however considerable gaps of knowledge concerning pathophysiology, treatments, and risk factors for developing a severe and sometimes fatal infection. Finding a marker that can predict the course of the Coronavirus disease 2019 (Covid‐19) would be of value for prioritizing patients in the healthcare system and for vaccination. Observational data indicates that the most common co‐morbidities in severe Covid‐19 are several of the diagnoses that make up the metabolic syndrome, that is, hypertension, diabetes mellitus type 2, obesity, and hyperlipidemia.^1^, ^2^, ^3^ Moreover, physical activity seems to reduce the risk of severe Covid‐19.^2^ Clinically, we observed that many patients with severe Covid‐19 had undiagnosed metabolic syndrome whereas the patients without metabolic imbalance hospitalized for non‐Covid related diagnosis seem to have mild or no signs of Covid‐19 when testing positive for SARS‐CoV‐2 during routine screening. This is supported by recent studies.^3^, ^4^ Therefore, we hypothesized that untreated diabetes mellitus might be associated with a higher risk of developing severe Covid‐19. Unfortunately, we lack a common biochemical marker for recognition of imbalance in the metabolic homeostasis. Dysglycemic metabolic disorders such as diabetes mellitus and pre‐diabetes, constitute themselves a pandemic with high prevalence and a rapidly increasing incidence of cases presenting to healthcare.^5^ The glycated hemoglobin (HbA1c) test is used as a reliable marker for glycemic status. The test is used to diagnose diabetes mellitus and to evaluate long‐term glycemic control. It has been proposed as a useful marker for early detection of insulin resistance, a major etiological factor for the development of the metabolic syndrome.^6^, ^7^

The aim of this exploratory study was to assess the association between chronic dysglycemia, diagnosed by analysis of HbA1c, and SARS‐CoV‐2 associated respiratory failure in hospitalized patients with a positive PCR‐test for SARS‐CoV‐2. We hypothesized that the presence of undiagnosed or insufficiently controlled chronic dysglycemia would be associated with the development of SARS‐CoV‐2 respiratory failure.

## 2 METHODS

The study was approved by the regional ethics committee in Stockholm (2020–01447, 2020–06969) with a waiver for informed consent and was conducted according to the Helsinki declaration.

### 2.1 Study population

We performed a retrospective observational study including adult patients (≥18 years) admitted between March 9, 2020 and September 6, 2020 (first wave) to the Karolinska University Hospital, Stockholm, Sweden. The hospital is a tertiary referral centre receiving trauma, neurosurgical and cardio‐thoracic patients from the larger Stockholm region, covering an urban area of approximately 2.5 million patients. In addition, a large number of patients with Covid‐19 respiratory failure have been admitted to the hospital´s medical wards or being transferred directly to the intensive care unit (ICU) from other hospitals during the study period. All patients were screened for SARS‐CoV‐2 on admission and some patients admitted with a primary diagnosis other than Covid‐19 tested positive for the virus on screening, many without displaying respiratory symptoms. All patients testing positive for severe acute respiratory syndrome coronavirus 2 (SARS‐CoV‐2) confirmed by polymerase chain reaction and with a HbA1c test obtained within 3 months prior to and including day of admission were included. We excluded patients who were admitted to ICU following extracorporeal membrane oxygenation therapy due to the risk of hemolysis, which may lower blood HbA1c, and patients admitted to hospital ward from ICU at another hospital.

### 2.2 Data collection

We included routine clinical data prospectively recorded in the Karolinska COVID‐19 quality database. Data were retrieved from available electronic patient data management systems (Centricity Critical Care [GE, Barrington IL] and Take Care [CompuGroup Medical, Koblenz, Germany]). Information regarding previous history of diabetes, diabetes‐type, diabetes treatment, previous history of hypertension, and antihypertensive treatment, statin use, corticosteroid treatment, and other immunosuppressive therapy was obtained by review of the patients’ medical records (notes, diagnosis codes and chronic medication). Information on the highest level of respiratory support (low‐flow oxygen, high‐flow oxygen, non‐invasive mechanical ventilation, or invasive mechanical ventilation) during hospital admission was collated from medical note review.

### 2.3 Operational definitions

We stratified patients into five groups according to preadmission glucose control (HbA1c strata recommended by the World Health Organization) and history of diabetes: (a) no diabetes (HbA1c <42 mmol/mol and no history of diabetes); (b) prediabetes (HbA1c 42–47 mmol/mol and no history of diabetes); (c) unknown diabetes (HbA1c ≥48 mmol/mol and no history of diabetes; (d) controlled diabetes (HbA1c <52 mmol/mol); (e) uncontrolled diabetes (HbA1c ≥52 mmol/mol).

### 2.4 Outcomes

The primary outcome was development of respiratory failure associated with SARS‐CoV‐2 infection as determined by the treating physicians. The treating physicians’ assessment of the presence of and cause for respiratory failure was obtained by careful review of clinical notes, ICD‐10 diagnosis codes and discharge summaries. SARS‐CoV‐2 associated respiratory failure was ruled out by the treating clinician when the patient had no respiratory symptoms or when respiratory failure was caused by conditions other than SARS‐CoV‐2 (e.g., bacterial pneumonia, pulmonary edema, pneumothorax, or invasive mechanical ventilation due to acute neurological injury) and was reversed by targeted treatment of the underlying cause.

SARS‐CoV‐2 associated respiratory failure will forthwith be referred to as respiratory failure.

### 2.5 Statistical analysis

Categorical data are presented as numbers and percentages and compared using the chi‐square test. Continuous data are presented as median with interquartile range and compared using the Mann–Whitney U test. The association between chronic glucose control (no diabetes, prediabetes, unknown diabetes, controlled diabetes, and uncontrolled diabetes) and HbA1c strata (<42, 42–47, 48–51, and ≥52), respectively, and primary outcome was assessed using separate multivariable logistic regression analyses. The analyses were adjusted for age, sex, and body mass index (BMI). In a sensitivity analysis we also adjusted for baseline variables with a *p* value < .1 on univariable analysis. Goodness of fit and model discrimination were determined using the Hosmer‐Lemeshow test and area under the receiver operating curve (ROC) respectively. All analysis was performed using STATA SE 14.2 and 15. A two‐sided *p*‐value of < .05 was considered statistically significant.

## 3 RESULTS

**FIGURE 1:**
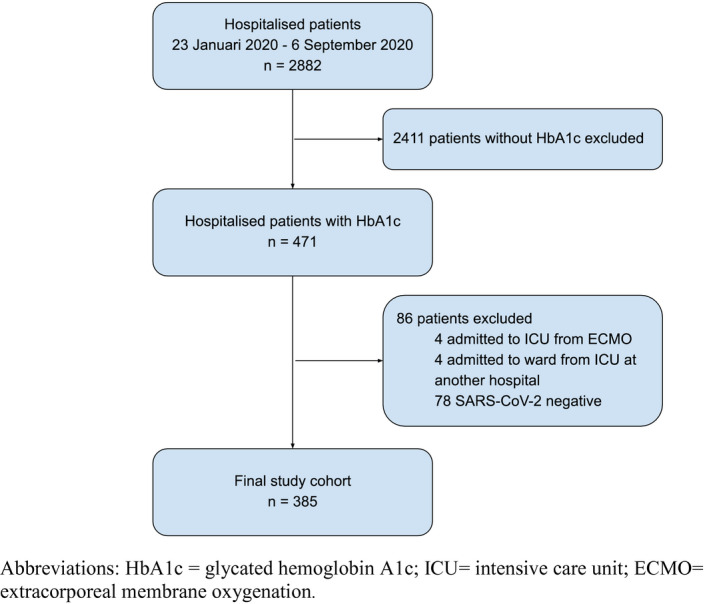
Patient selection. HbA1c, glycated hemoglobin A1c; ICU, intensive care unit; ECMO, extracorporeal membrane oxygenation

**FIGURE 2:**
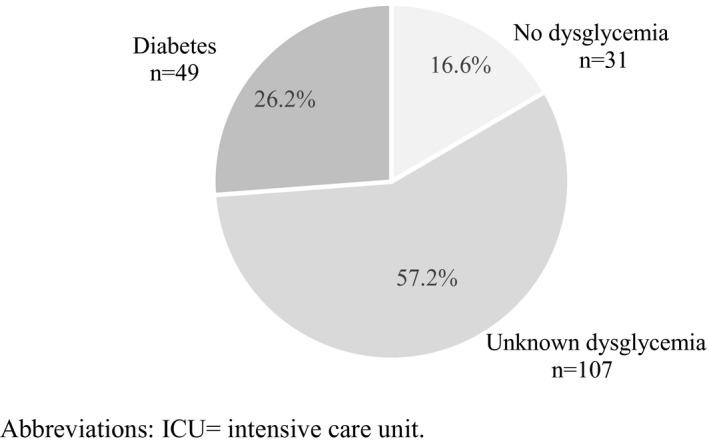
The proportion of patients with dysglycemia admitted to ICU due to SARS‐CoV‐2 associated respiratory failure, *n* = 187. ICU, intensive care unit

The selection of study patients is presented in Figure 1. We included 385 patients in the final analysis. Overall, 299 (78%) patients exhibited respiratory failure and, 86 patients (22%) did not. Baseline characteristics of patients with and without respiratory failure are compared in Table 1. Age and sex distributions were similar between groups. Patients with respiratory failure had higher median BMI (28 [IQR 25–31] vs. 25 [IQR 23–30], *p* < .001) and higher median HbA1c (48 [IQR 43–64] vs. 37 [34–42], *p* < .001) compared to the patients without. The proportion of patients with prediabetes, unknown diabetes, or uncontrolled diabetes was higher among patients with respiratory failure (Table 1). Conversely, patients without respiratory failure were more likely to have normal glycemic status or controlled diabetes. On groupwise comparison of co‐morbidities, cerebrovascular disease was more frequent in the group without respiratory failure than in the group with respiratory failure (20.9% vs. 6.7%, *p* < .001). A detailed list of primary reasons for hospital admission in patients without respiratory failure is provided in Table S1. Among the patients admitted to the ICU with respiratory failure (*n* = 187), 156 patients (83.4%) had a HbA1c >42 mmol/mol, of which 49 (26.2%) patients had a medical history of diabetes mellitus (Figure 2). Admission to the ICU (60.9% vs. 26.7%, *p* < .001), invasive mechanical ventilation (60.9% vs. 26.7%, *p* < .001) and renal replacement therapy (14% vs. 1.2%, *p* < 0.001) were more frequent in the group with respiratory failure compared to the group without. ICU (13.9 days (IQR 7.5–23.0 days)) and hospital (16.5 days (IQR 8.0–31.5 days)) length of stay were both longer in the group with respiratory failure compared to the those without (2.7 days (IQR 0.9–15.1 days and 6.7days (IQR 3.2–14.0 days) respectively). Ninety‐day mortality was not statistically different between the groups (22.1% vs. 14.0%, *p* = 0.10) (Table 2). In multivariable logistic regression analysis using no diabetes as reference category, prediabetes (OR 14.41 [95% CI 5.27–39.43]), unknown diabetes (OR 15.86 [95% CI 4.55–55.36]) and uncontrolled diabetes (OR 17.61 [95% CI 5.77–53.74]) were associated with respiratory failure, independent of age, sex, and BMI (Table 3). Adding cerebrovascular disease to the model did not markedly change these associations (Table S2). Finally, in a separate multivariable logistic regression analysis, all levels of HbA1c ≥42 mmol/mol (vs. below 42 mmol/mol) were independently associated with increased risk of respiratory failure (Table S3). Notably, the odds ratio for increased risk of respiratory failure due to SARS‐CoV‐2 was not linearly associated with the HbA1c level, for example, the odds ratio was higher in the 42–47 mmol/mol group (OR 12.62 [95% CI 5.18–30.75]) compared to the 48–51 mmol/mol group (OR 6.45 [95% CI 2.39–17.35]), but highest in the patients with a HbA1c ≥ 52 mmol/mol (OR 16.85 [95% CI 6.53–43.49]), (Table S3).

**TABLE 1:** Baseline characteristics of SARS‐CoV‐2 positive hospitalized patients

Characteristic	No SARS‐CoV−2 associated respiratory failure (n = 86)	SARS‐CoV−2 associated respiratory failure (*n* = 299)	*p*‐value
Age (year)	61 (45–73)	60 (53–69)	.75
Male sex, n (%)	58 (67.4)	227 (75.9)	.11
BMI (kg/m^2^)	25 (23–30)	28 (25–31)	<.001
HbA1c (mmol/mol)	37 (34–42)	48 (43–64)	<.001
Chronic dysglycemia, *n* (%)			<.001
No chronic dysglycemia	56 (65.1)	54 (18.1)	
Prediabetes	6 (7.0)	82 (27.4)	
Unknown diabetes	5 (5.8)	63 (21.1)	
Controlled diabetes	13 (15.1)	23 (7.7)	
Uncontrolled diabetes	6 (7.0)	77 (25.8)	
Antidiabetic treatment in patients with known diabetes, *n* (%)			
No treatment	0/19 (0)	3/100 (3)	.44
Diet	0/19 (0)	8/100 (8.0)	.20
Oral antihyperglycemic agents	7/19 (36.8)	40/100 (40.0)	.80
Insulin	6/19 (31.6)	25/100 (25.0)	.55
Combination of oral antihyperglycemic agents and Insulin	6/19 (31.6)	24/100 (24.0)	.49
Comorbidity, *n* (%)			
Hypertension	42 (49.4)	148 (49.5)	.99
Renal disease	13 (15.1)	57 (19.1)	.40
Chronic pulmonary disease	11 (12.8)	52 (17.4)	.31
Myocardial infarction	13 (15.1)	36 (12.0)	.45
Congestive heart failure	12 (14.0)	39 (13.0)	.83
Peripheral vascular disease	8 (9.3)	14 (4.7)	.10
Cerebrovascular disease	18 (20.9)	20 (6.7)	<.001
Dementia	4 (4.7)	4 (1.3)	.06
Rheumatic disease	2 (2.3)	6 (2.0)	.86
Peptic ulcer disease	2 (2.3)	5 (1.7)	.69
Any malignancy, including lymphoma	10 (11.6)	20 (6.7)	.13
Liver disease	2 (2.3)	8 (2.7)	.86
Chronic drug use, *n* (%)			
RAAS‐blockers	31 (36.0)	95 (31.8)	.46
Beta‐receptor blockers	25 (29.1)	91 (30.4)	.81
Calcium channel blockers	21 (24.4)	65 (21.7)	.60
Statins	25 (29.1)	88 (29.5)	.93
Immunosuppression	6 (7.0)	22 (7.4)	.90
Steroids	9 (10.5)	27 (9.0)	.69
Treatment‐limitations, *n* (%)	13 (15.3)	40 (13.4)	.66

Abbreviations: BMI, body mass index; HbA1c, glycated hemoglobin A1c; RAAS, renin‐angiotensin‐aldosterone‐system.

Data are presented as median (IQR) for continuous measures, and *n* (%) for categorical measures.

**TABLE 2:** Interventions and outcomes in SARS‐CoV‐2 positive hospitalized patients

Variable	No SARS‐Cov−2 associated respiratory failure (*n* = 86)	SARS‐Cov−2 associated respiratory failure (*n* = 299)	*p*‐value
ICU‐admission, *n* (%)	23 (26.7)	187 (62.5)	<.001
Mechanical Ventilation, *n* (%)	23 (26.7)	182 (60.9)	<.001
Non‐invasive ventilatory support, *n* (%)	0 (0)	16 (5.4)	.25
High‐flow oxygen support, *n* (%)	0 (0)	14 (4.7)	.29
Low‐flow oxygen support, *n* (%)	47 (67.1)	84 (28.2)	<.001
Renal replacement therapy, *n* (%)	1 (1.2)	42 (14)	<.001
ICU length of stay (days)	2.7 (0.9–15.1)	13.9 (7.5–23.1)	<.001
Hospital length of stay (days)	6.7 (3.2–14.0)	16.5 (8.0–31.5)	<.001
90‐day mortality, *n* (%)	12 (14.0)	66 (22.1)	.10

Abbreviations: ICU, intensive care unit.

Data are presented as median (IQR) for continuous measures, and *n* (%) for categorical measures.

**TABLE 3:** Univariable and multivariable logistic regression analysis showing the association with SARS‐CoV‐2 induced respiratory failure

	Univariable		Multivariable^a^	
Variable	Odds Ratio (95% CI)	*p*‐value	Odds Ratio (95% CI)	*p*‐value
Male sex	1.52 (0.90–2.57)	.116	1.91 (0.97–3.80)	.06
Age, years	1.01 (0.99–1.02)	.542	0.99 (0.97–1.01)	.24
BMI, kg/m^2^	1.09 (1.03–1.16)	.005	1.06 (0.99–1.13)	.09
Chronic dysglycemia				
No chronic dysglycemia	1.00			
Prediabetes	14.17 (5.71–35.19)	<.001	14.41 (5.27–39.43)	<.001
Unknown diabetes	13.07 (4.88–34.97)	<.001	15.86 (4.55–55.36)	<.001
Controlled diabetes	1.83 (0.84–3.99)	.125	2.08 (0.84–5.15)	.11
Uncontrolled diabetes	13.31 (5.35–33.10)	<.001	17.61 (5.77–53.74)	<.001

Abbreviations: BMI, body mass index; CI, confidence interval.

^a^ Model area under the curve 0.83, Hosmer‐Lemeshow *p*‐value 0.77

## 4 DISCUSSION

We conducted a retrospective exploratory study to assess the association between chronic dysglycemia, determined by assessment of HbA1c and diabetes history, and respiratory failure in hospitalized SARS‐CoV‐2 positive patients. We found that prediabetes, unknown diabetes, and uncontrolled diabetes were independently associated with increased risk of developing respiratory failure when infected with SARS‐CoV‐2. In contrast, we found no significant association between well‐controlled diabetes and risk of SARS‐CoV‐2 disease progression.

### 4.1 Relationship with previous studies

The most common co‐morbidities for patients with Covid‐19 observed in this study (hypertension, diabetes mellitus, renal disease, chronic pulmonary disease, and cardiac diseases) are in line with previously published studies.^1^, ^8^, ^9^, ^10^ As evidence relating to Covid‐19 and diabetes mellitus continues to emerge, people with diabetes have been identified as being at increased risk of serious Covid‐19. The majority of the literature regarding diabetes and Covid‐19 is reported without any differentiation between the two major types of diabetes but refers predominantly to diabetes mellitus type 2. Barron et. al. found an association between diabetes mellitus type 1 and poor Covid‐19 outcome. However, the patients at particular risk were older and burdened with co‐morbidities.^11^ However, our study included only 12 patients with diabetes mellitus type 1 and only one of them, a patient on immunosuppressive therapy, required ICU‐admission and mechanic ventilation. The different pathophysiologic processes underlying the metabolic disturbances in diabetes mellitus type 1 and type 2 warrants further investigation by diabetes type and Covid‐19 status. In addition, only a few studies have considered patients with previously unknown chronic dysglycemia or different levels of premorbid glycemic control among those individuals with an established diabetes diagnosis.

Congruent with other recent studies, we found a high incidence of prediabetes (HbA1c 42–48 mmol/mol) or diabetes (HbA1c > 48 mmol/mol) in patients with severe Covid‐19.^12^, ^13^, ^14^ In our study, prediabetes and undiagnosed diabetes as well as a poor glycemic control in patients with known diabetes mellitus constituted 74% of the patients with respiratory failure. In the same group of patients with respiratory failure, 62.5% of the patients were admitted to the ICU, of whom 61% were mechanically ventilated and 14% were supported with renal replacement therapy, indicating a severe course of the infection. Interestingly, our finding that normal glycemic status or well‐controlled diabetes in patients infected with SARS‐CoV‐2 was not associated with respiratory failure is supported by results from Smith et al. In a single‐center study, they found an association between dysregulated glucose metabolism and severe Covid‐19.^4^

Although patients with diseases constituting the metabolic syndrome are at high risk for severe Covid‐19, the link between SARS‐CoV‐2 and dysglycemia is not yet fully understood. Susceptibility to infection and a dysregulated immune response caused by diabetes,^15^ viral induced insulin resistance^16^ or a direct virus‐induced damage to endocrine cells of pancreas^17^ have all been proposed to contribute to the increased disease severity observed in these patients when infected with SARS‐CoV‐2. Another possible link could be the entry point for the virus, the angiotensin converting enzyme 2 (ACE2) receptor.^18^ After entering the cells, SARS‐CoV‐2 has been proposed to downregulate ACE2 creating an imbalance of the renin‐angiotensin‐aldosterone hormonal system (RAAS) with increased angiotensin II levels. Insulin resistance, a central component in the pathophysiology of the metabolic syndrome,^19^ has already been linked to an inappropriately overactive RAAS in several studies. In these studies, blocking RAAS in subjects with hypertension and/or cardiovascular disease improved insulin sensitivity and reduced incidence of new onset diabetes mellitus type 2.^20^ HbA1c elevation observed in the majority of patients in this study with severe Covid‐19 is likely to reflect a chronic underlying metabolic imbalance and may in part explain these patient´s vulnerability when infected with SARS‐CoV‐2. Pulmonary and systemic hypertension, hypertriglyceridemia, insulin resistance, kidney failure, and thromboembolic events are clinical features common to both severe COVID‐19 infection and the metabolic syndrome. Although occurring over very different times perspectives; rapidly over days or weeks in COVID‐19 but over a period of years in metabolic syndrome. Thus, one possible link between metabolic imbalance and SARS‐CoV‐2 could be that when a person with latent chronic metabolic imbalance becomes infected with SARS‐CoV‐2, the virus degrades ACE2, RAAS overactivation increases and the process of metabolic syndrome may accelerate, generating an *acute on chronic metabolic syndrome* situation. This is supported by a recent review demonstrating that RAAS‐blockers may be protective for Covid‐19 patients with hypertension. Furthermore, it may indicate that patients with underdiagnosed or insufficiently treated diseases of the metabolic syndrome may be at risk for severe Covid‐19.^21^

### 4.2 Study implications

Our findings imply that identification of patients with chronic dysglycemia and quantification of their premorbid control is important as patients with undiagnosed dysglycemia or poor diabetes control are at particular risk of developing respiratory complications when infected with SARS‐CoV‐ 2. Our findings also imply that that HbA1c measurement identifies a significant proportion of SARS‐CoV‐2 positive patients with prediabetes or previously unknown diabetes.

Early in the Covid‐19 pandemic, it was noted that patients with obesity were at higher risk for severe Covid‐19.^9^, ^22^ In the present study, we demonstrate that HbA1c seems to be another valuable marker for identifying patients at risk of severe Covid‐19. Early identification of patients at risk for severe Covid‐19 through an HbA1c test may prevent a severe course of the disease by targeted vaccination or by early specific treatment. Moreover, long‐term measures should of cause be taken to improve the metabolic status of the patients, in parallel with vaccination and treatment of covid‐19.

### 4.3 Strengths and limitations

Our study has several strengths. We assess the impact of prediabetes and unknown diabetes on the risk of developing severe Covid‐19. By comprehensive review of medical records, we were able to make a detailed assessment of several components of the metabolic syndrome and a thorough evaluation of SARS‐CoV‐2 infected patient at risk for Covid‐19 disease, reducing the risk for misclassification.

Our study has some limitations. HbA1c was not consecutively analyzed in all patients admitted to our hospital, which implies a risk of selection bias. Furthermore, we lack data of blood transfusion prior to HbA1c testing, which could display false low values of the test. Moreover, detailed data regarding respiratory failure using results of blood gas analysis and level of supplementary oxygen were not available in all patients. Additionally, the confidence intervals in our analyses of chronic dysglycemia and HbA1c were wide. However, the point estimates for prediabetes, unknown diabetes, and uncontrolled diabetes were also high indicating that a type I error is unlikely. Although well‐accounted for, residual confounding can never be completely ruled out in this study design. Finally, the single center design limits generalizability.

### 4.4 Conclusions

In our cohort of hospitalized, SARS‐CoV‐2 positive patients with available preadmission HbA1c, the presence of prediabetes, undiagnosed diabetes, and uncontrolled diabetes was associated with a markedly elevated risk of respiratory failure. We suggest that HbA1c could be used to detect patients at risk of developing severe Covid‐19 and using this information to prioritize vaccination and to guide monitoring and care at hospital admission. Our results support further investigation of the pathophysiological link between chronic dysglycemia, metabolic imbalance, and severe Covid‐19.

## CONFLICT OF INTERESTS

None of the authors have any conflict of interests.

## AUTHOR CONTRIBUTIONS

SR, MJF, and JM contributed to the design of the study. SR, JM, and FCJ collected patient data. SR, JM, CRS and EL performed and contributed to data analysis. The first draft of the manuscript was written by SR, MJF and JM. All authors commented on previous versions of the manuscript. All authors read and approved the final manuscript for publication.

## Supporting information

Table S1‐S3
