# Supplementary material for: Chronic dysglycemia and risk of SARS‐CoV‐2 associated respiratory failure in hospitalized patients
Source: Acta Anaesthesiol Scand. 2021 Oct 11;66(1):48–55. doi: 10.1111/aas.13982 (PMC8653023; doi:10.1111/aas.13982)
Supplement: Supplementary file 1 — Table S1‐S3 [file AAS-66-48-s001.docx]

Table S1. Admission diagnoses in patients with no SARS-CoV-2 associated respiratory failure

| **Diagnoses** | **ICU** | **Medical/surgical ward** |
| --- | --- | --- |
|  | n=23 | n=63 |
| **Trauma:** |  |  |
| Multitrauma | 10 | 7 |
| Traumatic brain injury | 3 | 0 |
| **Cardiac:** |  |  |
| Myocardial infarction/ Coronary syndrome | 0 | 12 |
| Cardiac arrest | 3 | 0 |
| Arrythmias/ congestive heart failure | 1 | 3 |
| **Pulmonary:** |  |  |
| Pneumothorax/ pulmonary bleeding | 0 | 2 |
| **Cerebral:** |  |  |
| Cerebrovascular diseases | 2 | 13 |
| **Vascular:** |  |  |
| Ischaemia, peripheral | 0 | 2 |
| **Infections:** |  |  |
| Pneumonia, bacterial | 1 | 0 |
| Urosepsis | 1 | 3 |
| Catheter related infections | 0 | 1 |
| Chemo induced neutropen sepsis | 0 | 1 |
| **Surgery** | 1 | 1 |
| **Complications from surgery** | 1 | 4 |
| **New onset diabetes mellitus** | 0 | 3 |
| **Deterioration in general condition/ dehydration** | 0 | 10 |
| **In-hospital hemodialysis** | 0 | 1 |
| Abbreviations:ICU=intensive care unit. | | |

Table S2. Univariable and multivariable logistic regression analysis showing the association with SARS-Cov-2 induced respiratory failure.

|  | **Univariable** | | **Multivariable*** | |
| --- | --- | --- | --- | --- |
| **Variable** | **Odds Ratio (95% CI)** | **p-value** | **Odds Ratio (95% CI)** | **p-value** |
| Male sex | 1.52 (0.90-2.57) | 0.116 | 2.13 (1.06-4.28) | 0.034 |
| Age, years | 1.01 (0.99-1.02) | 0.542 | 1.00 (0.97-1.02) | 0.727 |
| BMI, kg/m^2^ | 1.09 (1.03-1.16) | 0.005 | 1.05 (0.99-1.13) | 0.113 |
| Chronic dysglycemia |  |  |  |  |
| No chronic dysglycemia | 1.00 |  |  |  |
| Prediabetes | 14.17 (5.71-35.19) | < 0.001 | 16.00 (5.64-45.37) | < 0.001 |
| Unknown diabetes | 13.07 (4.88-34.97) | < 0.001 | 13.37 (3.81-46.94) | < 0.001 |
| Controlled diabetes | 1.83 (0.84-3.99) | 0.125 | 2.05 (0.80-5.24) | 0.135 |
| Uncontrolled diabetes | 13.31 (5.35-33.10) | < 0.001 | 16.16 (5.24-49.86) | < 0.001 |
| Cerebrovascular disease | 0.27 (0.14-0.54) | < 0.001 | 0.23 (0.02-0.61) | 0.003 |
| Abbreviations: CI = confidence interval; BMI = body mass index.  *Model area under the curve 0.84, Hosmer-Lemeshow p-value 0.91 | | | | |

Table S3. Univariable and multivariable logistic regression analysis showing the association with SARS-Cov-2-induced respiratory failure.

|  | **Univariable** | | **Multivariable*** | |
| --- | --- | --- | --- | --- |
| **Variable** | **Odds Ratio (95% CI)** | **p-value** | **Odds Ratio [95% CI\|** | **p-value** |
| Male sex | 1.52 (0.90-2.57) | 0.116 | 1.98 (1.00-3.92) | 0.049 |
| Age, years | 1.01 (0.99-1.02) | 0.542 | 0.98 (0.96-1.00) | 0.100 |
| BMI, kg/m^2^ | 1.09 (1.03-1.16) | 0.005 | 1.06 (0.99-1.13) | 0.085 |
| Chronic dysglycemia: |  |  |  |  |
| < 42 mmol/mol | 1.00 |  | 1.00 |  |
| 42-47 mmol/mol | 12.59 (5.61-28.25) | < 0.001 | 12.62 (5.18-30.75) | < 0.001 |
| 48-51 mmol/mol | 6.48 (2.69-15.62) | < 0.001 | 6.45 (2.39-17.35) | < 0.001 |
| ≥ 52 mmol/mol | 13.65 (6.33-29.47) | < 0.001 | 16.85 (6.53-43.49) | < 0.001 |
| Abbreviations: CI = confidence interval; BMI = body mass index; HbA1c= glycated hemoglobin A1c.  *Model area under the curve 0.82, Hosmer-Lemeshow p-value 0.94 | | | | |

# 
